# The multidrug efflux pump regulator AcrR directly represses motility in *Escherichia coli*

**DOI:** 10.1128/msphere.00430-23

**Published:** 2023-10-03

**Authors:** Jessica Maldonado, Barbara Czarnecka, Dana E. Harmon, Cristian Ruiz

**Affiliations:** 1 Department of Biology, California State University Northridge, Northridge, California, USA; Antimicrobial Development Specialists, LLC, Nyack, New York, USA

**Keywords:** AcrR, *flhDC*, motility, flagellum biosynthesis, multidrug efflux pump, AcrAB-TolC, *Escherichia coli*

## Abstract

Efflux and motility are two key biological functions in bacteria. Recent findings have shown that efflux impacts flagellum biosynthesis and motility in *Escherichia coli* and other bacteria. AcrR is known to be the major transcriptional repressor of AcrAB-TolC, the main multidrug efflux pump in *E. coli* and other *Enterobacteriaceae*. However, the underlying molecular mechanisms of how efflux and motility are co-regulated remain poorly understood. Here, we have studied the role of AcrR in direct regulation of motility in *E. coli*. By combining bioinformatics, electrophoretic mobility shift assays (EMSAs), gene expression, and motility experiments, we have found that AcrR represses motility in *E. coli* by directly repressing transcription of the *flhDC* operon, but not the other flagellum genes/operons tested. *flhDC* encodes the master regulator of flagellum biosynthesis and motility genes. We found that such regulation primarily occurs by direct binding of AcrR to the *flhDC* promoter region containing the first of the two predicted AcrR-binding sites identified in this promoter. This is the first report of direct regulation by AcrR of genes unrelated to efflux or detoxification. Moreover, we report that overexpression of AcrR restores to parental levels the increased swimming motility previously observed in *E. coli* strains without a functional AcrAB-TolC pump, and that such effect by AcrR is prevented by the AcrR ligand and AcrAB-TolC substrate ethidium bromide. Based on these and prior findings, we provide a novel model in which AcrR senses efflux and then co-regulates efflux and motility in *E. coli* to maintain homeostasis and escape hazards.

**IMPORTANCE:**

Efflux and motility play a major role in bacterial growth, colonization, and survival. In *Escherichia coli*, the transcriptional repressor AcrR is known to directly repress efflux and was later found to also repress flagellum biosynthesis and motility by Kim et al. (J Microbiol Biotechnol 26:1824–1828, 2016, doi: 10.4014/jmb.1607.07058). However, it remained unknown whether AcrR represses flagellum biosynthesis and motility directly and through which target genes, or indirectly because of altering the amount of efflux. This study reveals that AcrR represses flagellum biosynthesis and motility by directly repressing the expression of the *flhDC* master regulator of flagellum biosynthesis and motility genes, but not the other flagellum genes tested. We also show that the antimicrobial, efflux pump substrate, and AcrR ligand ethidium bromide regulates motility via AcrR. Overall, these findings support a novel model of direct co-regulation of efflux and motility mediated by AcrR in response to stress in *E. coli*.

## INTRODUCTION

Gram-negative bacteria possess multidrug efflux (MDR) pumps that are highly conserved within the species and between families (1 – 4). The role of MDR pumps in conferring multiple antibiotic resistance is well-studied, especially for AcrAB-TolC, which is a major MDR pump of *Escherichia coli* and other *Enterobacteriaceae* (3 – 6). AcrAB-TolC and its homologs can efflux most classes of antibiotics and other toxic compounds entering the cells from the outside, acting synergistically with the permeability barrier provided by the outer membrane of Gram-negatives to prevent the accumulation of antibiotics in these bacteria (3, 5).

Less understood are the biological roles of MDR pumps beyond the efflux of antibiotics or toxic compounds such as bile salts (1 – 7). Recent findings have shown that MDR pumps, especially AcrAB-TolC, have a broad impact on gene expression, metabolism, stress responses, signaling, virulence, colonization, motility, and other physiological processes (3 – 5, 7 – 22). However, the molecular mechanisms that allow MDR pumps to control and coordinate all these processes remain mostly unknown. Especially intriguing is the impact of efflux on motility, which is an important function that allows cells to colonize new environments, move toward nutrients, and escape hazards, among other functions (23, 24).

In *E. coli*, the impact of efflux on motility was first discovered in a mutant deleted for the *acrB* gene (9), which encodes for the substrate recognition and binding component of the AcrAB-TolC pump (3 – 5, 25 – 27). Deletion of *acrB* produced broad changes in gene expression, especially a very strong upregulation of nearly all flagellum biosynthesis and motility genes (9). Accordingly, this mutant showed an increase in swimming motility compared to the wild-type strain (9). This finding raised the question of how AcrAB-TolC, which is located in the cell envelope, can regulate the expression of flagellum biosynthesis, motility, and other genes. A later study in *E. coli* showed that deletion of the *acrAB* main transcriptional repressor *acrR* (9, 28) produced a similar upregulation of flagellum biosynthesis and motility genes (which was accompanied by increased flagella production), and a similar increase in swimming motility (29) to those we observed in the Δ*acrB* mutant (9). Changes in motility caused by efflux pumps or their regulators, either similar or opposite to those found in *E. coli*, have also been found in other bacteria such as *Salmonella enterica*, *Serratia marcescens*, or *Acinetobacter baumanii* (8, 16, 30 – 32). For example, while AcrR has been found to repress both efflux (9, 28) and motility (29) in *E. coli*, Thota and Chubiz (32) found that, in *S. enterica*, the transcriptional regulators MarA, SoxS, Rob, and RamA, known to activate the expression of efflux genes, instead repress flagellar gene expression and motility. These findings indicate that the interplay between efflux and motility is both important and specific for the biology of a broad number of bacteria. However, the molecular mechanisms of how such interplay occurs are not well-understood yet.

Here, we have examined the role of AcrR in regulating swimming motility in *E. coli*. AcrR is a TetR-family transcriptional regulator composed of a C-terminal ligand-binding domain and an N-terminal DNA-binding domain (33). The ligands that control the activity of AcrR have been found to be both exogenous antimicrobials known to be AcrAB-TolC substrates, i.e., ethidium bromide, as well as cellular metabolites, i.e., polyamines (9, 34, 35). Regarding its target genes, AcrR was initially identified as the local transcriptional repressor of the *acrAB* operon (28), but was later found to also directly regulate the expression of the *acrAB* activators SoxS and MarA (36), as well as the putrescine degradation gene *puuA* and the spermidine efflux operon *mdtUJI* (35). However, it remained unknown whether AcrR also directly regulates flagellum biosynthesis genes and motility in *E. coli*, and which motility genes are direct targets of this regulator, or whether AcrR indirectly regulates flagellum genes and motility by regulating efflux or other target genes. Here, we report that AcrR is a direct regulator of the *flhDC* operon, which encodes for the master regulator of flagellum biosynthesis and motility genes, but does not directly regulate the *fliE*, *fliLMNOPQR* or *fliDST* genes/operons. We also show that such direct regulation of swimming motility by AcrR allows *E. coli* to synergistically respond to ethidium bromide not only by increasing efflux [activating *acrAB* expression (35)], but also by increasing swimming motility. These findings support a model in which AcrR senses the accumulation of antimicrobials or cellular metabolites normally effluxed by the AcrAB-TolC multidrug efflux pump and then directly co-regulates efflux and motility to increase efflux and to escape from these compounds.

## RESULTS

### Bioinformatics analyses suggest that the AcrR transcriptional repressor directly regulates flagellum biosynthesis and motility genes

Prior findings have shown that inactivation of the transcriptional repressor AcrR (Δ*acrR::kan* mutant) results in a strong upregulation of about 50 flagellum biosynthesis and motility genes and increased flagella production, alongside with an increase in swimming (0.3% agar) but not swarming (0.6%) motility (29). These findings alongside with the identification in the *flhDC* promoter of three 10 bp fragments with partial overlap with the known 24 bp AcrR-binding site in the *acrAB* promoter (34), led to the suggestion that the effects of AcrR on motility might be mediated by *flhDC* (29). The FlhD_4_C_2_ complex is the known master regulator that activates the expression of flagellum biosynthesis and motility genes (37, 38).

To further investigate the role of AcrR as a direct regulator of motility in *E. coli*, we performed a whole-genome bioinformatics search of promoters regions with predicted AcrR-binding sites using the known 24 bp AcrR-binding site in the *acrAB* promoter (34) and the Colibri (http://genolist.pasteur.fr/Colibri/) search tool. We have recently found that AcrR directly regulates the putrescine degradation gene *puuA* and the spermidine efflux operon *mdtUJI*, whose promoters contain one predicted AcrR-binding site with 9 mismatches and two sites with 10 mismatches, respectively, compared to the AcrR-binding site in the *acrAB* promoter (35). Therefore, to maximize the finding of potential AcrR target genes, we focused our analysis on hits with promoter regions that contained at least one predicted AcrR-binding site with 11 or less mismatches compared to the known AcrR site in the *acrAB* promoter. We found that four motility genes/operons fulfilled these criteria: *flhDC* (motility master regulator; class I), *fliE* (component of the hook-basal body complex; class II), *fliLMNOPQR* (components of the flagellar motor switch and flagellar export apparatus; class II), and *fliDST* (FliD, flagellar filament capping protein; FliST: chaperones of the flagellar export system; class III).

**Fig 1:**
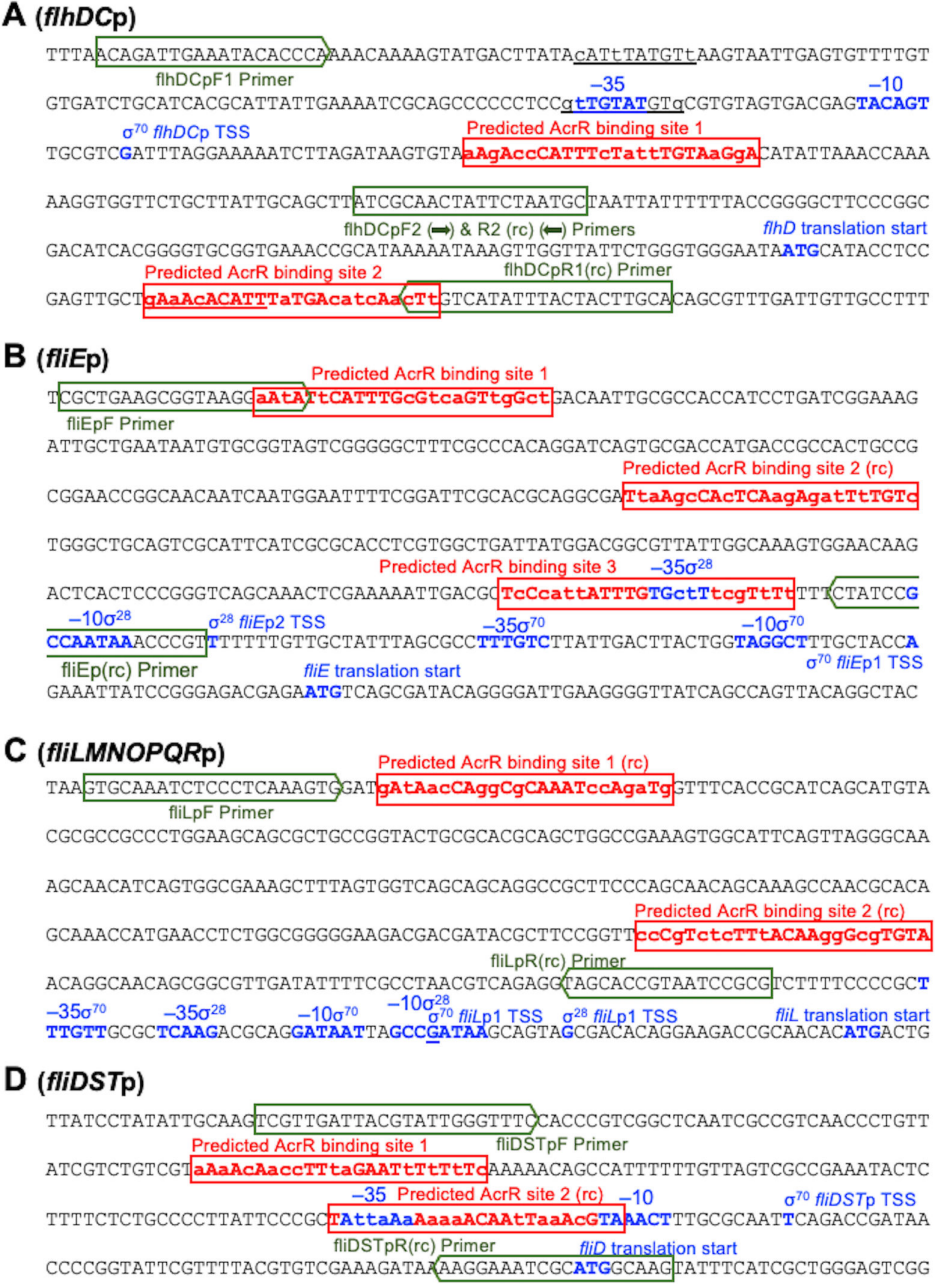
Predicted AcrR-binding sites in the four flagellum biosynthesis and motility genes/operons identified as direct target candidates of the AcrR repressor. (**A**) Sequence of the σ^70^-dependent promoter region of the *E. coli* master regulator of flagellar biosynthesis and motility *flhDC* operon (*flhDC*p). The −35 and −10 sequences, the transcriptional start site (TSS), and the *flhD* translational start site, are highlighted in blue lettering. The two predicted AcrR-binding sites in the *flhDC* promoter identified in this study, which had 10 and 11 mismatches, respectively, compared to the 24 bp AcrR-binding site in the *acrAB* promoter [5′-TACATACATTTGTGAATGTATGTA (34)], are indicated in red lettering, with mismatches shown as lowercase letters. The three partial (10 bp) AcrR-binding sites identified by Kim et al. (29) are indicated as underlined lettering. The forward primers and reverse complementary (rc) sequences for the reverse primers used to amplify the *flhDC* full promoter and promoter fragments used in EMSA (Fig. 2A) are indicated as green boxes. (B−D) Sequences of the *fliE*, *fliLMNOPQR*, and *fliDST* promoter regions (*fliE*p, *fliLMNOPQR*p, and *fliDST*p), respectively. Predicted AcrR-binding sites, promoter elements, and primers used for EMSA are labeled in the same manner as described above for *flhDC*. Predicted AcrR-binding sites labeled as (rc) denote that the sequence and mismatches shown correspond to the reverse complementary sequence (opposite strand) of the AcrR-binding site (5′-TACATACATTCACAAATGTATGTA). *fliE*p contained three predicted AcrR-binding sites with 11, 12, and 12 mismatches, respectively, compared to the AcrR-binding site in the *acrAB* promoter. *fliMNOPQR*p and *fliDST*p each contained two predicted AcrR-binding sites with 12 and 11 mismatches, for sites 1 and 2 in each promoter, respectively.

We then computationally analyzed more in-depth the promoter regions of the four flagellum biosynthesis and motility genes identified as potential AcrR direct targets (Fig. 1). For the *flhDC* promoter region, in addition to the three 10 bp partial AcrR sites previously identified (29), which were found upstream of the −35 element, overlapping the −35 element, and downstream of the *flhD* translation start codon, respectively, we found two full-size (24 bp) predicted AcrR-binding sites (Fig. 1A). Site 1 was located downstream of the transcriptional start site and contained 10 mismatches compared to the AcrR site in the *acrAB* promoter. Site 2 had 11 mismatches compared to the AcrR site in the *acrAB* promoter and was located downstream of the *flhD* translation start codon and overlapping with one of the 10 bp partial sites identified by Kim et al. (29) (Fig. 1A). For the *fliE* promoter region (Fig. 1B), we identified three full-size predicted AcrR-binding sites with 11, 12, and 12 mismatches, respectively, compared to the AcrR site in the *acrAB* promoter. Site 3 overlapped the σ^28^ −35 element, and the other two sites were located significantly upstream. For the *fliLMNOPQR* promoter (Fig. 1C), we found two full-size predicted AcrR-binding sites with 12 and 11 mismatches, respectively, both located significantly upstream of the σ^70^ and σ^28^ −35 elements. For the *fliDST* promoter (Fig. 1D), we identified two full-size predicted AcrR-binding sites with 12 and 11 mismatches, respectively, with site 2 overlapping both the σ^70^ −35 (fully) and −10 (partially) elements, and site 1 being located upstream.

**Fig 2:**
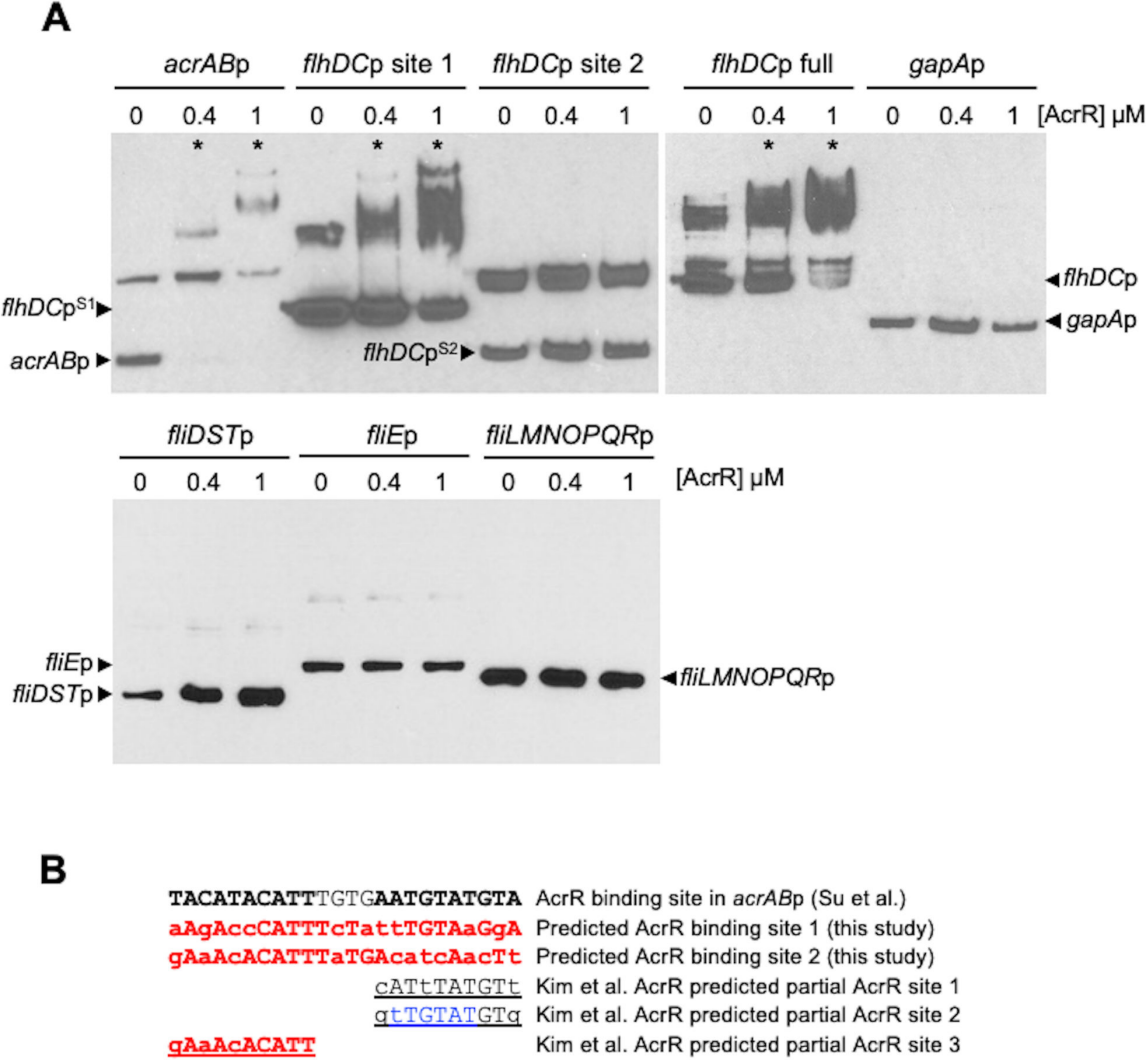
AcrR directly binds to the *flhDC* but not the *fliE*, *fliMNOPQR*, or *fliDST* promoter regions *in vitro*. (**A**) Top panels, EMSA showing *in vitro* binding of purified AcrR at both 0.4 and 1 µM to the *acrAB* promoter (positive control), the *flhDC* promoter fragment containing predicted AcrR-binding site 1 (*flhDC*p^S1^, amplification product obtained using primers flhDCpF1 and flhDCpR2), and the full *flhDC* promoter (*flhDC*p; obtained by using primers flhDCpF1 and flhDCpR1). Purified AcrR did not bind to the *flhDC* promoter fragment containing only predicted AcrR-binding site 2 (*flhDC*p^S2^, amplification product obtained using primers flhDCpF2 and flhDCpR1) or the *gapA* promoter (*gapA*p, negative control). (**A**) Bottom panel, EMSA showing that purified AcrR did not bind to the *fliE, fliMNOPQR or fliDST* promoters. (**A**) Top and bottom panels, the EMSA results shown are representative of at least three independent assays. For the *acrAB*p, *flhDC*p, *flhDC*p^S1^ *, flhDCp* ^S2^, *fliDST*, and *fliE* promoter fragments, two bands were observed in the absence of AcrR (0 µM AcrR, DNA only). Of them, the lower band corresponds to the expected size of the DNA fragment and is labeled with a black arrow next to the name of the promoter fragment tested. The higher band is a nonspecific band that appears both in the absence and presence of AcrR. For the *acrAB* promoter, *flhDC* full promoter, and *flhDC* promoter fragment containing AcrR site 1 (*flhDC*p^S1^), lanes in which one or more DNA-shifted bands can be observed because of binding of purified AcrR are labeled as “*.” The presence of shifted bands of different sizes may indicate binding of one versus two AcrR dimers to that DNA fragment. (**B**) Alignment of the AcrR-binding site in the *acrAB* promoter (34)—which includes two 10-bp inverted repeats (shown in bold lettering) separated by 4 bp—, the two predicted full AcrR-binding sites in the *flhDC* promoter identified in this study, and the three partial AcrR-binding sites in the *flhDC* promoter identified by Kim et al. (29). For each full or partial predicted site, mismatches compared to the AcrR-binding site in the *acrAB* promoter are indicated in lowercase letters. Greater overall conservation, and especially in the second 10-bp inverted repeat, can be observed for the full predicted AcrR-binding site 1 in *flhDC*p compared to the full predicted AcrR-binding site 2. This difference may explain why AcrR was found to bind to the promoter fragment with only site 1 (*flhDC*p^S1^), but not to the promoter fragment with only site 2 (*flhDC*p^S2^), in the above EMSA (**A**).

### AcrR represses motility by directly repressing the *flhDC* operon, which encodes for the master regulator of flagellum biosynthesis and motility genes, *in vitro* and *in vivo*, and does not directly regulate *fliE*, *fliLMNOPQR*, or *fliDST in vitro*

We next used EMSAs to determine whether purified AcrR directly binds to the promoter regions of the four flagellum and motility genes/operons identified as potential direct targets of AcrR (Fig. 2A). For each of these four genes/operons, we used promoter fragments that included all their predicted AcrR-binding sites (the primer regions used to amplify each promoter are indicated in green boxes in Fig. 1). In agreement with previous findings (35), AcrR bound to the *acrAB* promoter (positive control), but not the *gapA* promoter (negative control). Importantly, we found that AcrR also binds to the *flhDC* promoter region (Fig. 2A). This finding is consistent with the increased expression of flagellum biosynthesis genes and increased motility previously found in the Δ*acrR::kan* mutant (29), and with the presence of the two aforementioned predicted binding sites for AcrR in the *flhDC* promoter (Fig. 1A). We also found that AcrR does not directly bind to the *fliE*, *fliLMNOPQR* or *fliDST* promoters (Fig. 2A). Overall, these findings indicate that AcrR regulates motility in *E. coli* by directly regulating transcription of the *flhDC* master regulator of flagellum biosynthesis and motility. However, we cannot discard that AcrR might also directly regulate other flagellum and motility genes not identified here as potential AcrR targets.

The AcrR site in the *acrAB* promoter contains two 10-bp inverted repeats (Fig. 2B, in bold) connected by a 4-bp spacer (34). AcrR has been suggested to bind to this site as a dimer of dimers, with each dimmer binding opposite to each other on the double-stranded DNA site, and each monomer in a dimer binding to each one of the two 10-bp inverted repeats (33, 34). Of the two full predicted AcrR-binding sites identified here in the *flhDC* promoter, site 1 was the most conserved. In fact, site 1 contains only 10 mismatches compared to the AcrR site in the *acrAB* promoter, and those mismatches are evenly distributed leaving two inverted repeats each with 60% conservation and thus likely remain functional (Fig. 2B). On the contrary, site 2 contains 11 mismatches, which results in a 70% conserved left inverted repeat but a 30% poorly conserved right inverted repeat (Fig. 2B). We hypothesize that this lack of conservation on the right inverted repeat of site 2 may hinder binding of its corresponding AcrR monomer.

Interestingly, when two different fragments of the *flhDC* promoter region were tested, we found that AcrR binds to the fragment that contained the full predicted AcrR site 1 identified here [plus two of the partial sites identified by Kim et al. (29)], but not to the fragment that contained only the full predicted AcrR site 2 (Fig. 2A). These findings are in agreement with the greater overall conservation, including two likely functional inverted repeats, described above for site 1 compared to site 2. However, it is possible that both sites 1 and 2 [and perhaps the additional partial AcrR-binding sites identified by Kim et al. (29)] are important for regulation of *flhDC* by AcrR *in vivo*, or that prior binding of AcrR to site 1 contributes to binding of AcrR to site 2.

**Fig 3:**
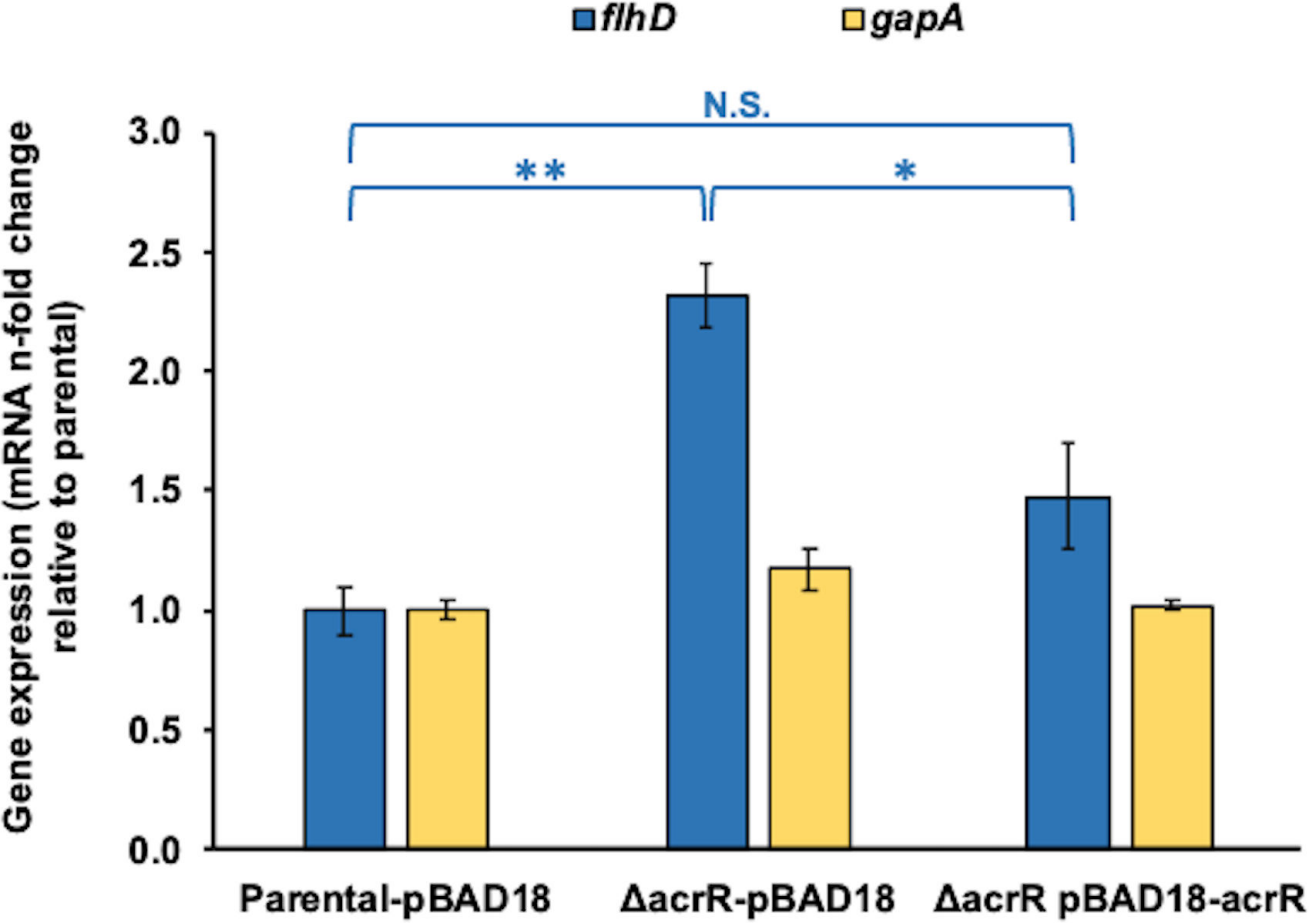
AcrR regulates the expression of the *flhDC* operon *in vivo*. The expression of *flhD* and the control gene *gapA* were measured by RT-qPCR using RNA extracted from mid-exponential phase cultures in LB with 50 µg/mL kanamycin that were induced for 1 h with 0.4% arabinose. Experiments were performed using three to five biological replicates each with two RT and three qPCR technical replicates. The data are presented as average ±SEM (*n* = 3–5) and are shown as the *n*-fold change in the expression of *flhD* or *gapA* of each strain normalized to that of the parental strain. No statistically significant differences were found between any of the strains for the control gene *gapA* (statistics not shown in the figure for clarity). For *flhD*, statistically significant differences between strains are indicated as ** (*P* < 0.001) or * (*P* < 0.05); and lack of statistically significant differences is indicated as N.S. The expression of *flhD* was significantly increased in the Δ*acrR* mutant compared to the parental strain, whereas complementation of this mutant using the pBAD18-Kan-*acrR* plasmid reduced *flhD* expression to a level similar to that in the parental.

**Fig 4:**
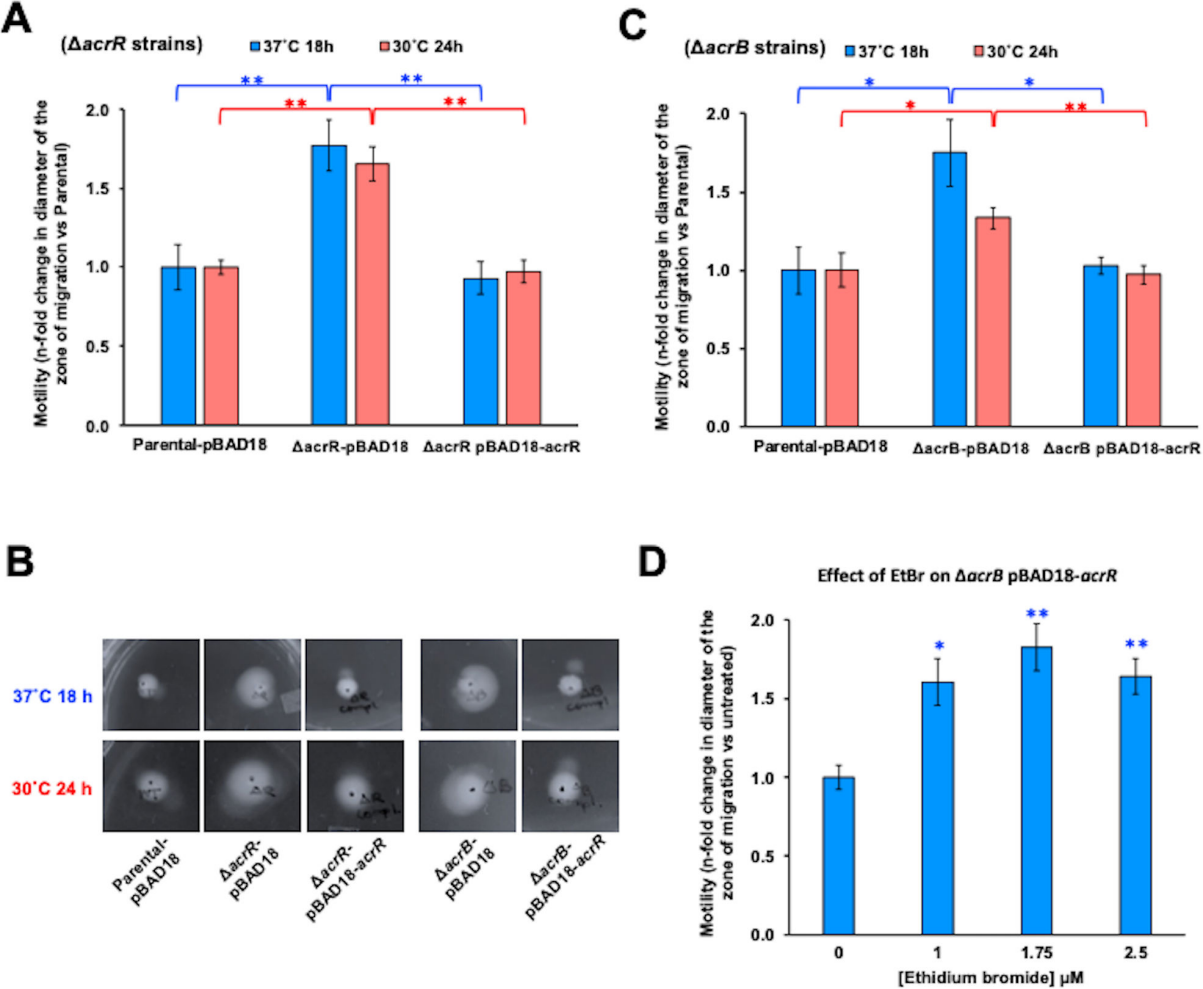
AcrR represses swimming motility in *E. coli* at 37°C and 30°C. (A–C) Motility was measured as the diameter of the zone of migration in mm using LB 0.35% agar plates supplemented with 0.4% arabinose and 50 µg/mL kanamycin after incubation at 37°C for 18 h (blue bars) or 30°C for 24 h (red bars). All experiments were performed using four to six biological replicates. The data are presented as average ±SEM (*n* = 4–6) and are shown as the *n*-fold change in the diameter of the zone of migration of each strain normalized to that of the parental strain, which was 5.3 ± 0.5 mm at 37°C for 18 h, and 8.4 ± 0.4 mm at 30°C for 24 h. Statistically significant differences between the Δ*acrR* or Δ*acrB* mutants compared to the parental strain (all containing the empty pBAD18-Kan plasmid), or between each mutant containing the pBAD18-Kan plasmid and the same mutant with the pBAD18-Kan-*acrR* plasmid, are indicated as ** (*P* < 0.001) or * (*P* < 0.05). No statistically significant differences were found between the parental-pBAD18-Kan and the Δ*acrR* or Δ*acrB* mutant strains containing the pBAD18-Kan-*acrR* plasmid (statistics not shown in the figure for clarity). (**A**) The Δ*acrR* mutant showed significant increased motility at both temperatures compared to the parental strain. Complementation of the Δ*acrR* mutation using the pBAD18-Kan-*acrR* plasmid restored the motility of this mutant to parental levels. (**B**) Representative pictures of the swimming motility results obtained for the strains tested in panels (A and C). (**C**) The Δ*acrB* mutant showed significant increased motility at both temperatures compared to the parental. Overexpression of the *acrR* gene using the pBAD18-Kan-*acrR* plasmid restored the motility of this mutant to the parental level at both 37°C and 30°C. (**D**) Addition of increasing concentrations of the AcrR ligand ethidium bromide (EtBr) prevents the swimming motility reduction caused by overexpressing *acrR* in the Δ*acrB* mutant. Statistically significant differences between the untreated (0 µM) and ethidium bromide treatments are indicated as ** (*P* < 0.01) or * (*P* < 0.05).

After finding that AcrR directly binds to the *flhDC* promoter, we next studied the effect of AcrR on the expression of the *flhDC* operon using real-time quantitative PCR (RT-qPCR) assays (Fig. 3). The expression of *flhDC* increased by 2.3-fold in the Δ*acrR* mutant compared to the parental strain (Fig. 3), in agreement with our EMSA results, and the 2.3- to 2.6-fold increase found for this operon in the Δ*acrR::kan* mutant by Kim et al. (29) using a microarray. As expected, complementation of the Δ*acrR* mutant by expressing *acrR* from the pBAD18-Kan-*acrR* plasmid reduced the expression of the *flhDC* operon in this mutant to a level close to that in the parental strain (Fig. 3), although there was a residually higher, but non-statistically significant, *flhD* expression in the complemented strain that we speculate was caused by *acrR* expression from the pBAD18-Kan-*acrR* plasmid having only been induced for one hour. This hypothesis is supported by the fact that full complementation of the Δ*acrR* mutant was observed in the motility experiments described below (Fig. 4A), which involve much longer induction times. Overall, our gene expression findings further support our bioinformatics and EMSA results, and thus the role of AcrR as a direct transcriptional repressor of *flhDC*.

### AcrR represses swimming motility in *E. coli* at both 37°C and 30°C

We next studied whether AcrR regulates swimming motility in *E. coli* at 37°C (human host temperature), as well as at 30°C (environmental temperature) for comparison with prior motility assays performed at 30–33°C (9, 29) (Fig. 4A and B). We found that deletion of *acrR* significantly increased swimming motility in *E. coli* at both 37°C and 30°C, by 1.8-fold and 1.7-fold, respectively. Overall, our motility results with the Δ*acrR* mutant at both 37°C and 30°C are in agreement with the increased swimming motility found at 33°C in a Δ*acrR::kan* mutant by Kim et al. (29), confirming that such increased motility was caused by the lack of *acrR*, and not by the *kan* cassette. Moreover, complementation of the Δ*acrR* mutant by expressing *acrR* from the pBAD18-Kan-*acrR* plasmid significantly decreased motility in this mutant, completely restoring its motility to the parental levels at both temperatures (Fig. 4A). These complementation experiments, not included in the study of Kim et al. (29), further confirm the specific effect of *acrR* in repressing motility in *E. coli*. In addition, our motility results are in agreement with our findings that AcrR directly binds to the promoter of the flagellum biosynthesis and motility regulator *flhDC* operon and represses its expression (Fig. 1 to 3), further supporting the role of AcrR as a direct repressor of swimming motility.

**Fig 5:**
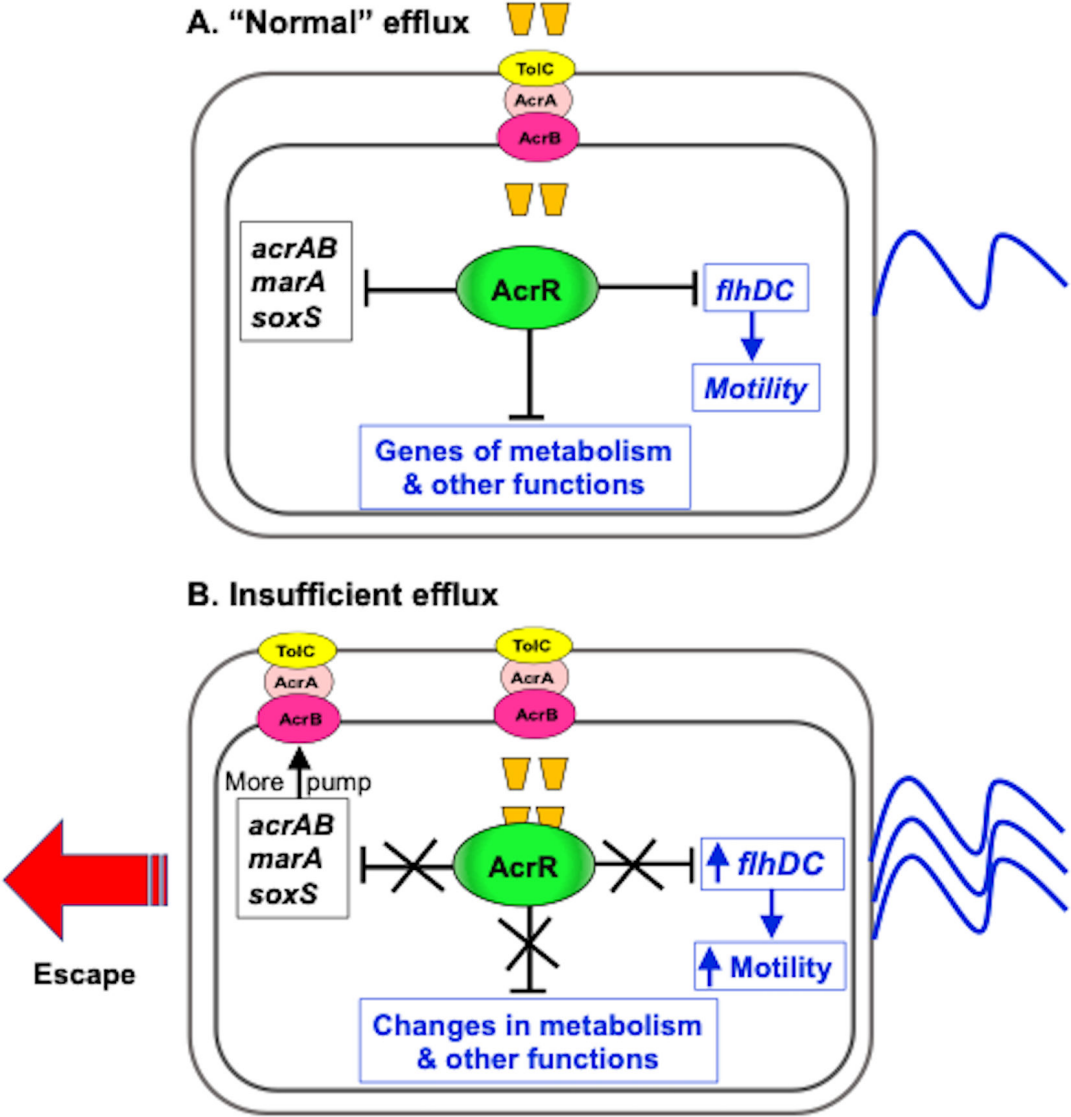
Model of the role of AcrR in co-regulation of efflux and motility in *E. coli*. We hypothesize that the two main functions of the AcrAB-TolC pump are to efflux antimicrobials such as ethidium bromide (1 – 7) and to efflux cellular metabolites (9, 10, 17, 18), whereas the transcriptional repressor AcrR acts as the main sensor and gene-expression effector of this pump. In normal conditions (**A**), efflux would prevent the accumulation of antimicrobials or cellular metabolites, and AcrR would keep at basal levels the expression of *acrAB* (9), its transcriptional activators MarA and SoxS (9), the *puuA* and *mdtJI* genes for polyamine detoxification and efflux (35), and the *flhDC* motility master regulator (Fig. 1 to 3). (**B**) When efflux is insufficient or the AcrAB-TolC pump is deleted, cells would accumulate antimicrobials such as ethidium bromide and/or cellular metabolites such as polyamines that bind to and inactivate AcrR (35). These cellular metabolites (9, 10, 17, 18), which would be themselves AcrAB-TolC substrates, or intermediates, end-products or by-products of these substrates, may either function as siderophores, signaling molecules, be toxic, or cause cellular stress because their accumulation would disrupt the normal metabolic flow of cells. Thus, inactivation of AcrR by antimicrobials or cellular metabolites would derepress *acrAB*, *marA*, and *soxS*, as previously observed (9), to increase the production of the AcrAB-TolC pump and thus facilitate the efflux of these compounds. Inactivation of AcrR would also depress genes involved in metabolism and other functions such as the polyamine detoxification and efflux genes *puuA* and *mdtJI* (35) to contribute to maintaining homeostasis. Finally, inactivation of AcrR would also lead to increased expression of *flhDC* (Fig. 1 to 3), which in turn would increase the expression of flagellum biosynthesis and motility genes. Overexpression of these genes would ultimately increase motility (Fig. 4) and facilitate the escape of *E. coli* cells from antimicrobials and/or the accumulated cellular metabolites by moving to a different environment.

Finally, we tested whether AcrR played a role in the increased swimming motility previously found by Ruiz and Levy (9) when the AcrAB-TolC efflux pump was inactivated by deletion of the *acrB* gene (Fig. 4B and C). Given the role of AcrR in directly regulating *flhDC* and swimming motility reported here (Fig. 1 to 4), and that cellular metabolites that accumulate in the Δ*acrB* mutant have been suggested to bind to and inactivate AcrR (9, 17), we hypothesized that inactivation of AcrR by cellular metabolites contributes to the increased motility previously found in the Δ*acrB* mutant. Thus, we also hypothesized that overexpression of AcrR would compensate such metabolic inactivation and restore motility in the Δ*acrB* mutant. We found that the Δ*acrB* mutant showed an increase in swimming motility of 1.8-fold at 37°C and of 1.3-fold at 30°C compared to the parental strain (Fig. 4B and C), in agreement with our prior findings at 30°C for this mutant (9). Interestingly, overexpression of *acrR* using the pBAD18-Kan-*acrR* plasmid restored motility in the Δ*acrB* mutant at both temperatures (Fig. 4C). This finding strongly supports our hypothesis that inactivation of AcrR by cellular metabolites contributes to the increase in motility found when AcrAB-TolC efflux pump is inactivated, and supports the model summarized in Fig. 5 and discussed below. To further test the hypothesis that ligands that bind to and inactivate AcrR contribute to the increased motility found in the Δ*acrB* mutant, we studied the effect of adding ethidium bromide to the Δ*acrB* mutant containing pBAD18-Kan-*acrR* plasmid (Fig. 4D). Ethidium bromide is an exogenous molecule not produced or metabolized by *E. coli*, and has recently been found to bind to and inactivate AcrR (35). We used low ethidium bromide concentrations to minimize its toxic effects in the Δ*acrB* mutant. Consistent with our hypothesis, addition of ethidium bromide to the Δ*acrB*+pBAD18-Kan-*acrR* mutant made this mutant hypermotile again, thus preventing the effects of having additional AcrR copies in the motility of this mutant (Fig. 4D).

## DISCUSSION

Efflux and motility are major contributors to the ability of bacteria to grow, colonize, and survive toxic compounds or environmental changes (1 – 6, 8 – 24). Interestingly, recent findings have shown that both functions are interconnected in bacteria such as *E. coli*, *S. enterica*, *S. marcescens*, and *A. baumanii* (8, 9, 16, 30, 31). In *E. coli*, Ruiz and Levy (9) found that deletion of the *acrB* gene (Δ*acrB*), which encodes for the inner membrane component that recognizes and binds to the substrates of the AcrAB-TolC efflux pump, causes upregulation of the *acrAB* operon, as well as a strong upregulation of about 50 flagellum biosynthesis and motility genes in cells grown in lysogeny broth (LB). Accordingly, the Δ*acrB* mutant was hypermotile when compared to the parental strain in swimming motility assays performed in LB 0.3% agar plates at 30°C (9).

Interestingly, a later study by Kim et al. (29) revealed that a similar upregulation of flagellum biosynthesis and motility genes, and a similar increase in swimming motility occurs, along with increased flagella production, when the *acrAB* transcriptional repressor *acrR* gene was deleted (Δ*acrR::kan* mutant). Such findings, together with the identification in the *flhDC* promoter of three putative 10-bp fragments with partial overlap with the known 24-bp AcrR binding site, whereas no predicted AcrR sites were identified in the *fliAZ* promoter, suggested that the effects of AcrR on motility might be mediated by *flhDC* (29). The FlhD_4_C_2_ complex is the known master regulator that activates the expression of flagellum biosynthesis and motility genes, whereas *fliA* encodes for the σ^28^ factor that controls the expression of a subset of motility genes and is downstream of the FlhD_4_C_2_ master regulator in the regulatory cascade of flagellum biosynthesis and motility genes (37, 38). However, it was still unknown whether AcrR directly regulates the expression of *flhDC* and/or other flagellum biosynthesis and motility genes in *E. coli*, or whether AcrR indirectly regulates flagellum genes and motility by regulating efflux and/or other target genes. To address this gap in knowledge, we have examined the role of AcrR as a direct regulator of flagellum biosynthesis and motility genes, as well as its role in the increased motility previously found in both Δ*acrB* and Δ*acrR* mutants.

First, we performed a genome-wide bioinformatics search of motility genes with promoters that contained predicted AcrR-binding sites using the known 24-bp AcrR-binding site in the *acrAB* promoter (34). We identified four flagellum biosynthesis and motility genes/operons—*flhDC*, *fliE*, *fliDST*, and *fliLMNOPQR*—whose promoter regions contained at least one full (24 bp) predicted AcrR-binding site (Fig. 1). We next tested for direct binding of purified AcrR to these four promoter regions by EMSA and found that AcrR only binds to the *flhDC* promoter region (Fig. 2A). This promoter region contains two predicted AcrR-binding sites with 10 and 11 mismatches, respectively, compared to the AcrR binding site in the *acrAB* promoter. In contrast, the *fliE*, *fliDST*, and *fliLMNOPQR* promoter regions each contain one predicted site with 11 mismatches plus one or two predicted sites with 12 mismatches (Fig. 1). Overall, our bioinformatics and EMSA findings indicate that the broad overexpression of about 50 flagellum biosynthesis genes and motility genes and increased motility previously found in the Δ*acrR* mutant (29) is the result of AcrR being a direct repressor of the motility master regulator *flhDC* operon. However, we do not discard that, in addition to its role as a *flhDC* regulator, AcrR might also directly regulate other motility genes/operons not identified here as potential AcrR targets.

To further investigate the role of AcrR in repressing motility by directly repressing *flhDC* expression, we combined bioinformatics, *in vitro*, *in vivo* gene expression and motility assays (Fig. 1 to 4). We first investigated the two potential full-size (24 bp) AcrR-binding sites identified in the *flhDC* promoter region. Site 1, located downstream of the transcriptional start site, has 10 mismatches compared to the known AcrR site in the *acrAB* promoter and maintains two 60% conserved inverted repeats. Site 2 has 11 mismatches compared to the known AcrR site in the *acrAB* promoter, including a 70% conserved left inverted repeat and a 30% poorly conserved right inverted repeat (Fig. 1A and 2B). Using EMSA to test for direct binding of purified AcrR protein to two different fragments of the *flhDC* promoter region, we found that besides AcrR binding to and shifting the full *flhDC* promoter region, AcrR was also able to bind to and shift the promoter fragment containing site 1 but not site 2 (Fig. 2A). On the contrary, no binding of AcrR was found for the promoter fragment containing only site 2 (Fig. 2A). Combined, these findings strongly support the role of AcrR as a direct regulator of the *flhDC* operon, and suggest that the more conserved site 1 might be the primary binding site for AcrR.

To further test the hypothesis that AcrR directly represses the *flhDC* operon, we performed *in vivo* gene expression and swimming motility assays comparing a parental strain, with the Δ*acrR* mutant, and the Δ*acrR* mutant complemented with the *acrR* gene cloned in an inducible plasmid. Deletion of *acrR* significantly increased the expression of the *flhDC* operon and motility at both 37°C and 30°C (Fig. 3, 4A and B), which is consistent with previous findings (29). In addition, we show for the first time that complementation of the Δ*acrR* mutant by overexpression of a*crR* from a plasmid reduces *flhDC* expression and motility at both temperatures down to the parental levels. These results are in agreement with our findings that the *flhDC* promoter region contains two predicted full AcrR-binding sites and that purified AcrR directly binds to this promoter region. Overall, these findings indicate that AcrR regulates motility by acting as a direct transcriptional repressor of the *flhDC* master regulator of flagellum biosynthesis and motility genes.

Finally, we examined the role of AcrR in the interplay between the AcrAB-TolC multidrug efflux pump and swimming motility in *E. coli*. Considering that AcrR represses *acrAB* transcription, earlier findings that deletion of *acrB* or *acrR* produced a similar overexpression of flagellum biosynthesis and motility genes and a similar increase in swimming motility (9, 29) might seem counterintuitive. However, several factors may contribute in explaining these findings. Both the AcrAB-TolC pump and flagella are powered by the proton motive force (PMF). PMF consumption by AcrAB-TolC has been suggested to impact its fitness contributions (39), and might also impact flagella function given that the speed of the flagellar motor varies with the PMF (40). Thus, the increase in swimming motility found in the Δ*acrB* mutant might be the result of an increase in PMF available for flagella rotation in this mutant. However, given that deletion of the *acrR* repressor leads to overexpression of the AcrAB-TolC pump and thus less available PMF, it would be expected that motility would decrease in the Δ*acrR* mutant, which is the opposite of what Kim et al. (29) and this study (Fig. 4A and B) have observed. Moreover, changes in PMF would not explain why the Δ*acrB* and Δ*acrR* mutants both show a similar strong overexpression of flagellum biosynthesis and motility genes. Instead, based on our results here showing that AcrR is a direct repressor of *flhDC*, we hypothesize that the similar flagellum gene expression and motility changes observed in both Δ*acrB* and Δ*acrR* mutants may occur because cellular metabolites that accumulate in the Δ*acrB* mutant can function as ligands that bind to and inactivate AcrR. Such ligand inactivation of AcrR in the Δ*acrB* mutant would derepress the expression of *flhDC*, which would explain why this mutant behaves as the Δ*acrR* mutant. Consistent with this hypothesis, we indeed found that overexpression of *acrR* from a plasmid reduced motility in the Δ*acrB* mutant down to parental levels at both 37°C and 30°C (Fig. 4C); whereas the addition of the AcrR ligand ethidium bromide prevented such effect by AcrR in the Δ*acrB* mutant (Fig. 4D). However, we do not discard that, in addition of *flhDC* upregulation caused by ligand-mediated inactivation of AcrR, changes in PMF may also contribute to the increased motility of the Δ*acrB* mutant.

Overall, these and prior findings portray a broader and more complex role of AcrR beyond being the local repressor of *acrAB*, and suggest that AcrR plays a major role in sensing stress caused by the accumulation of AcrAB-TolC substrates and coordinating efflux, metabolism and motility in response to such stress. This model is detailed in Fig. 5 and is based on three major premises: The first premise is that besides its known role in removing exogenous antimicrobials such as antibiotics, bile salts or ethidium bromide (1 – 7), the AcrAB-TolC pump also plays a role in effluxing cellular metabolites. These metabolites would be effluxed because they themselves, or their intermediates, end-products or by-products, may function as siderophores, signaling molecules, be toxic, and/or disrupt the normal metabolic flow of cells when they accumulate. A metabolic role of AcrAB-TolC is supported by its role in exporting enterobactin (18); the increased *acrAB* expression previously found in metabolic mutants (9, 10); the altered expression of many metabolic genes found in Δ*acrB* and Δ*acrR* mutants (9, 29); and the global changes in the intracellular and extracellular metabolite profile we found in the Δ*acrB* and Δ*acrR* mutants by untargeted metabolomics (17). Among other changes, these included a strong accumulation of amino acids (e.g., lysine) and tricarboxylic acid cycle intermediates in the Δ*acrB* mutant (17).

The second premise is that AcrR senses the accumulation of antimicrobials and cellular metabolites that are AcrAB-TolC substrates, or their metabolic precursors or derivatives, because some of these compounds can function as ligands that bind to and inactivate AcrR (Fig. 5). This premise is supported by the findings that AcrR was required for the increased *acrAB* expression found in the Δ*acrB* mutant and that this role was dependent on changes in its activity, not its expression (9); the finding that three exogenous compounds with antimicrobial activity and known to be effluxed by AcrAB-TolC (ethidium bromide, rhodamine 6G, and proflavine), bind *in vitro* to both AcrR and AcrB with a similar dissociation constant (34, 41); and our recent findings that ethidium bromide and three cellular metabolites (polyamines such as the lysine-derivative cadaverine) directly bind to and inactivate AcrR, thus derepressing the *acrAB* promoter (35), and presumably other AcrR-regulated genes.

The third premise is that AcrR is a direct regulator that, once it has sensed the accumulation of AcrAB-TolC substrates such as ethidium bromide, or cellular metabolites that may disrupt normal metabolic flow or be toxic, directly coordinates a broad cell response that involves efflux, metabolic and motility changes to cope with the accumulation of these substrates and/or their precursors or derivatives (Fig. 5). This premise is supported by the well-known role of AcrR as a direct repressor of the *acrAB* operon (3 – 6, 28, 35); its role as a regulator SoxS and MarA expression (36), which are direct activators of the expression of *acrAB* and other genes involved in coping with antibiotics and other toxic molecules (4, 5, 42, 43); the recent finding that AcrR directly regulates polyamine detoxification and efflux genes (35); and the findings reported here that AcrR is a direct repressor of the master motility regulator *flhDC*.

In conclusion, this manuscript combined bioinformatics, EMSA, gene expression, and motility assays to reveal that AcrR regulates motility in *E. coli* by acting as direct transcriptional repressor of the *flhDC* operon, which encodes for the master regulator of flagellum biosynthesis and motility genes. To our knowledge, this is the first report of AcrR directly regulating genes unrelated to efflux or detoxification, which contributes to potentially redefine AcrR as a central regulator of a global stress response regulon. The results reported here and prior findings support a model in which AcrR senses the accumulation of antimicrobials or cellular metabolites effluxed by the AcrAB-TolC multidrug efflux pump, and then co-regulates efflux, metabolism, and motility to synergistically contribute to maintaining homeostasis and adapting to environmental hazards.

## MATERIALS AND METHODS

### Growth conditions, strains, and plasmids

*E. coli* strains were routinely grown at 37°C on LB (10 g/L tryptone, 5 g/L yeast extract, and 10 g/L NaCl) or LB agar (LB with 15 g/L agar) supplemented with 50 µg/mL kanamycin (LB-Kan) to maintain the pBAD18 (pBAB18-Kan) or pBAD18-*acrR* (pBAD18-Kan-*acrR*) plasmids. The strains and plasmids used in this study are listed in Table 1.

**TABLE 1:** Bacterial strains and plasmids used in this study

Strain/plasmid	Description	Source/reference
*E. coli* strains		
BW25113	(Parental) F^–^ λ^–^ Δ(*araD–araB)567* Δ*lacZ4787*(::*rrnB-3*) *rph-1* Δ(*rhaD–rhaB)568 hsdR514*	CGSC, Keio collection (44)
DH5α-pBAD18-Kan	Source of plasmid pBAD18-Kan	CGSC
DH7311	BW25113 ∆*acrR*	(35)
CR5000	BW25113 ∆*acrB*	(9)
BC7307	BW25113+pBAD18-Kan	This study
JM7312	DH7311 (BW25113 ∆*acrR*)+pBAD18-Kan	This study
JM7314	DH7311 (BW25113 ∆*acrR*)+pBAD18-Kan-*acrR*	This study
JM7316	CR5000 (BW25113 ∆*acrB*)+pBAD18-Kan-*acrR*	This study
JM7317	CR5000 (BW25113 ∆*acrB*)+pBAD18-Kan	This study
Plasmids		
pET21(+)-*acrR*	pET21(+)-derivative; AmpR, IPTG-inducible vector to overexpress AcrR with a 6x-His C-terminal tag for AcrR protein purification	(35)
pBAD18-Kan	Arabinose-inducible vector (*araBAD* promoter), KanR, used to clone *acrR* and as “empty plasmid” in complementation experiments	(45)
pBAD18-Kan-*acrR*	pBAD18-Kan derivative; KanR, *acrR* gene cloned after the *araBAD* promoter and an RBS, used in complementation experiments	This study

Plasmid pBAD18-Kan-*acrR* was constructed as follows. First, the *acrR* gene from the parental *E. coli* BW25113 strain was amplified by PCR using the Thermo Fisher Scientific (Waltham, MA, USA) DreamTaq polymerase as recommended by the manufacturer, a *T*_*m*_ of 60°C, and primers acrRclF (5′-GATCGAGCTCAGGAGGCGAACAT**ATG**GCACGAAA; SacI site underlined, translation start codon of a*crR* in bold) and acrRclR (5′-GATCCTGCAGGTCAGATTCAGGG**TTA**TTCG, PstI site underlined, complement sequence of the *acrR* translation stop codon in bold). After amplification, the PCR product was column-purified, digested with SacI and PstI (New England Biolabs, Ipswich, MA, USA), and ligated into pBAD18-Kan linearized with the same enzymes and gel-purified to generate plasmid pBAD18-Kan-*acrR*. Correct cloning of *acrR* was confirmed by plasmid isolation using of the Plasmid Miniprep kit from Zymo Research (Irvine, CA, USA) followed by Sanger sequencing at Laragen Inc (Culver City, CA, USA). Plasmids pBAD18-Kan or pBAD18-Kan-*acrR* were then electroporated into the parental strain *E. coli* BW25113 or its Δ*acrR* or Δ*acrB* mutant derivatives to generate the strains listed in Table 1 used for gene expression and motility complementation experiments.

### Bioinformatics analysis to identify potential AcrR-binding sites in the *flhDC* promoter and other flagellum biosynthesis and motility genes

The sequence of the known 24-bp AcrR-binding site in the *acrAB* promoter (*acrAB*p) [5′-TACATACATTTGTGAATGTATGTA (34)] and search tool of Colibri (http://genolist.pasteur.fr/Colibri/) were used to perform an initial whole-genome search of genes in *E. coli* that contain potential AcrR-binding sites and thus might be directly regulated by AcrR, focusing on flagellum biosynthesis and motility genes. The promoter regions of the four flagellum/motility genes/operons identified as candidate direct targets of AcrR (*flhDC*p, *fliE*p, *fliMNOPQR*, and *fliDST*p) were then computationally analyzed more in-depth using MEGA X v.11 software (46) to identify all potential AcrR-binding sites, the major promoter features, and design primers to test for AcrR binding to these promoters in the EMSA experiments described below.

### AcrR purification and electrophoretic mobility shift assays

AcrR protein expression and purification experiments were performed growing *E. coli* DH7293 containing plasmid pET21(+)-*acrR* in LB medium supplemented with 100 µg/mL ampicillin, using 100 µM IPTG to induce AcrR expression, followed by French Pressure cell lysis and AcrR purification using a NTA Sepharose column, as previously described (35).

EMSAs were performed as described by Harmon and Ruiz (35). Briefly, assays were performed using purified AcrR at a final concentration of 0 (DNA-only), 0.4, and 1 µM; PCR-generated, gel-extracted, promoter fragments; and the LightShift Chemiluminescent EMSA Kit from Thermo Fisher Scientific. The primers used to generate the *acrAB* (positive control) and *gapA* (negative control) promoter fragments are described elsewhere (35). Primers flhDCpF1 (5′-ACAGATTGAAATACACCCA) and flhDCpR1 (5′-TGCAAGTAGTAAATATGACAAG) were used to amplify the full 407-bp *flhDC* promoter region containing all predicted AcrR-binding sites (Fig. 1A). Primers flhDCpF1 and flhDCpR2 (5′-GCATTAGAATAGTTGCGAT) were used to generate the 256-bp *flhDC* promoter fragment containing predicted AcrR-binding site 1; and primers flhDCpF2 (5′-ATCGCAACTATTCTAATGC) and flhDCpR1 were used to generate the 170-bp *flhDC* promoter fragment containing predicted AcrR-binding site 2. Primers fliEpF (5′-CGCTGAAGCGGTAAGGAAT) and fliEpR (5′- ACGGGTTTATTGGCGGATAG) were used to amplify the 367-bp *fliE* promoter region containing all predicted AcrR-binding sites (Fig. 1B). Primers fliLpF (5′- GTGCAAATCTCCCTCAAAGTG) and fliLpR (5′- CGCGGATTACGGTGCTA) were used to amplify the 344-bp *fliLMNOPQR* promoter region containing all predicted AcrR-binding sites (Fig. 1C). Primers fliDpF (5′- TCGTTGATTACGTATTGGGTTTC) and fliDpR (5′- CTTGCCATGCGATTTCCTT) were used to amplify the 250-bp *fliDST* promoter region containing all predicted AcrR-binding sites (Fig. 1D).

### Gene expression experiments

The expression of the *flhDC* operon, measured as *flhD* mRNA, was determined by reverse transcription followed RT-qPCR as previously described (9, 47) with the following modifications. First, strains BC7307 (parental + pBAD18-Kan), JM7312 (Δ*acrR* + pBAD18-Kan), and JM7314 (Δ*acrR* + pBAD18-Kan-*acrR*), were grown for 20 h in LB-Kan medium at 37°C with agitation at 200 rpm. Next, cultures were subcultured 1:1,000 in fresh LB-Kan and incubated until they reached an OD_600 nm_ of 0.1, before adding arabinose at a 0.4% final concentration to induce the expression of *acrR* from the pBAD18-Kan-*acrR* plasmid. Cultures were then incubated for approximately 1 h until they reached mid-exponential phase (OD_600 nm_ = 0.4). Next, cultures were treated with RNAprotect Bacteria reagent (Qiagen, Valencia, CA, USA) to stabilize their RNA, followed by RNA extraction using the Qiagen RNAeasy Mini Kit and the Qiagen on-column RNAse-free DNAse kit. The purity and concentration of the extracted RNAs were then determined using a Nanodrop 2000 (Thermo Fisher Scientific).

RNAs were then diluted to a concentration of 50 µg/mL in RNase-free water before reverse transcribing 200 ng of each RNA using the Thermo Fisher Scientific RT Invitrogen SuperScript IV First-Strand Synthesis System Kit and random hexamers, with RNAse H treatment, according to the manufacturer’s specifications. RT minus reactions with water instead of reverse transcriptase were used as controls to confirm the lack of DNA contamination in the purified RNA samples.

After reverse transcription, *flhD* and *gapA* levels were quantified using 5 ng of total cDNA, 300 nM each of gene-specific forward and reverse primer, and the Applied Biosystems SYBR Green PowerUp Master Mix and QuanStudio 3 thermocycler (Thermo Fisher Scientific) as recommended by the manufacturer, using gene-specific standard plots for absolute quantification. qPCR gene-specific primers for *flhD* were: RTflhDF 5′-GCTATGTTTCGTCTCGGCATAA and RTflhDR 5′-CGGAAGTGACAAACCAGTTGA (*T*_*m*_ = 63°C), and were designed using the PrimerQuest tool (qPCR with intercalating dyes parameters) from Integrated DNA Technologies (https://www.idtdna.com/Primerquest). Primers for *gapA*, which was used as our control gene because it is known not regulated by AcrR (29, 35), are described elsewhere (48). All experiments were conducted using three to five biological replicates (cultures/RNA extractions), each with two (RT reactions) and three (qPCR reactions) technical replicates.

### Motility experiments

Swimming motility experiments were performed as previously described by Ruiz and Levy (9) with the following modifications. First, strains BC7307 (parental + pBAD18-Kan), JM7312 (Δ*acrR* + pBAD18-Kan), JM7314 (Δ*acrR* + pBAD18-Kan-*acrR*), JM7317 (Δ*acrB* + pBAD18-Kan), and JM7316 (Δ*acrB* + pBAD18-Kan-*acrR*) were grown for 20 h in LB-Kan agar plates at 37°C. Next, a single representative colony of each strain was stabbed using a sterile toothpick onto semi-solid LB-Kan plates containing 0.35% agar (which made the semi-solid medium in plates incubated at 37°C less fragile than using 0.3%) and 0.4% arabinose to induce the expression of *acrR* from the pBAD18-Kan-*acrR* plasmid. Inoculated plates were incubated for 18 h (or 24 h in motility assays performed in the presence of ethidium bromide) at 37°C, or 24 h at 30°C, before measuring the diameter of the zone of migration in mm. All experiments were conducted using five to six biological replicates.

### Statistical analysis

For gene expression and swimming motility experiments, statistically significant differences between strains or treatments were determined by *t* test (two independent samples with equal variance, two-tailed distribution) using Microsoft Excel 2021 software.

## References

[1] Blanco P , Hernando-Amado S , Reales-Calderon JA , Corona F , Lira F , Alcalde-Rico M , Bernardini A , Sanchez MB , Martinez JL . 2016. Bacterial multidrug efflux pumps: much more than antibiotic resistance determinants. Microorganisms 4:14. doi:10.3390/microorganisms4010014 27681908 PMC5029519

[2] Martinez JL , Sánchez MB , Martínez-Solano L , Hernandez A , Garmendia L , Fajardo A , Alvarez-Ortega C . 2009. Functional role of bacterial multidrug efflux pumps in microbial natural ecosystems. FEMS Microbiol Rev 33:430–449. doi:10.1111/j.1574-6976.2008.00157.x 19207745

[3] Li X-Z , Elkins CA , Zgurskaya HI . 2016. Efflux-mediated antimicrobial resistance in bacteria: mechanisms, regulation and clinical implications. Springer Berlin Heidelberg, New York, NY. doi:10.1007/978-3-319-39658-3

[4] Du D , Wang-Kan X , Neuberger A , van Veen HW , Pos KM , Piddock LJV , Luisi BF . 2018. Multidrug efflux pumps: structure, function and regulation. Nat Rev Microbiol 16:523–539. doi:10.1038/s41579-018-0060-x 30002505

[5] Li XZ , Plésiat P , Nikaido H . 2015. The challenge of efflux-mediated antibiotic resistance in gram-negative bacteria. Clin Microbiol Rev 28:337–418. doi:10.1128/CMR.00117-14 25788514 PMC4402952

[6] Nishino K , Yamaguchi A . 2001. Analysis of a complete library of putative drug transporter genes in Escherichia coli. J Bacteriol 183:5803–5812. doi:10.1128/JB.183.20.5803-5812.2001 11566977 PMC99656

[7] Teelucksingh T , Thompson LK , Zhu S , Kuehfuss NM , Goetz JA , Gilbert SE , MacNair CR , Geddes-McAlister J , Brown ED , Cox G . 2022. A genetic platform to investigate the functions of bacterial drug efflux pumps. Nat Chem Biol 18:1399–1409. doi:10.1038/s41589-022-01119-y 36065018

[8] Webber MA , Bailey AM , Blair JMA , Morgan E , Stevens MP , Hinton JCD , Ivens A , Wain J , Piddock LJV . 2009. The global consequence of disruption of the AcrAB-TolC efflux pump in Salmonella enterica includes reduced expression of SPI-1 and other attributes required to infect the host. J Bacteriol 191:4276–4285. doi:10.1128/JB.00363-09 19411325 PMC2698494

[9] Ruiz C , Levy SB . 2014. Regulation of acrAB expression by cellular metabolites in Escherichia coli. J Antimicrob Chemother 69:390–399. doi:10.1093/jac/dkt352 24043404 PMC3886929

[10] Helling RB , Janes BK , Kimball H , Tran T , Bundesmann M , Check P , Phelan D , Miller C . 2002. Toxic waste disposal in Escherichia coli. J Bacteriol 184:3699–3703. doi:10.1128/JB.184.13.3699-3703.2002 12057966 PMC135154

[11] Rosner JL , Martin RG . 2009. An excretory function for the Escherichia coli outer membrane pore TolC: upregulation of marA and soxS transcription and rob activity due to metabolites accumulated in tolC mutants. J Bacteriol 191:5283–5292. doi:10.1128/JB.00507-09 19502391 PMC2725600

[12] Nishino K , Latifi T , Groisman EA . 2006. Virulence and drug resistance roles of multidrug efflux systems of Salmonella enterica serovar Typhimurium. Mol Microbiol 59:126–141. doi:10.1111/j.1365-2958.2005.04940.x 16359323

[13] Guérin F , Lallement C , Isnard C , Dhalluin A , Cattoir V , Giard J-C . 2016. Landscape of resistance-nodulation-cell division (RND)-type efflux pumps in Enterobacter cloacae complex. Antimicrob Agents Chemother 60:2373–2382. doi:10.1128/AAC.02840-15 26856831 PMC4808149

[14] Padilla E , Llobet E , Doménech-Sánchez A , Martínez-Martínez L , Bengoechea JA , Albertí S . 2010. Klebsiella pneumoniae AcrAB efflux pump contributes to antimicrobial resistance and virulence. Antimicrob Agents Chemother 54:177–183. doi:10.1128/AAC.00715-09 19858254 PMC2798511

[15] Lawler AJ , Ricci V , Busby SJW , Piddock LJV . 2013. Genetic inactivation of acrAB or inhibition of efflux induces expression of ramA. J Antimicrob Chemother 68:1551–1557. doi:10.1093/jac/dkt069 23493314 PMC3682690

[16] Wang-Kan X , Blair JMA , Chirullo B , Betts J , La Ragione RM , Ivens A , Ricci V , Opperman TJ , Piddock LJV , Davies JE , Shafer W , Bonomo R . 2017. Lack of AcrB efflux function confers loss of virulence on Salmonella enterica serovar Typhimurium. mBio 8:e00968-17. doi:10.1128/mBio.00968-17 28720734 PMC5516257

[17] Cauilan A , Ramos K , Harmon DE , Ruiz C . 2019. Global effect of the AcrAB-TolC multidrug efflux pump of Escherichia coli in cell metabolism revealed by untargeted metabolomics. Int J Antimicrob Agents 54:105–107. doi:10.1016/j.ijantimicag.2019.05.015 31108224

[18] Horiyama T , Nishino K . 2014. AcrB, AcrD, and MdtABC multidrug efflux systems are involved in enterobactin export in Escherichia coli. PLoS One 9:e108642. doi:10.1371/journal.pone.0108642 25259870 PMC4178200

[19] Aoki SK , Malinverni JC , Jacoby K , Thomas B , Pamma R , Trinh BN , Remers S , Webb J , Braaten BA , Silhavy TJ , Low DA . 2008. Contact-dependent growth inhibition requires the essential outer membrane protein BamA (YaeT) as the receptor and the inner membrane transport protein AcrB. Mol Microbiol 70:323–340. doi:10.1111/j.1365-2958.2008.06404.x 18761695 PMC2579741

[20] Thanassi DG , Cheng LW , Nikaido H . 1997. Active efflux of bile salts by Escherichia coli. J Bacteriol 179:2512–2518. doi:10.1128/jb.179.8.2512-2518.1997 9098046 PMC178997

[21] El Meouche I , Dunlop MJ . 2018. Heterogeneity in efflux pump expression predisposes antibiotic-resistant cells to mutation. Science 362:686–690. doi:10.1126/science.aar7981 30409883 PMC6343669

[22] Wang-Kan X , Rodríguez-Blanco G , Southam AD , Winder CL , Dunn WB , Ivens A , Piddock LJV . 2021. Metabolomics reveal potential natural substrates of AcrB in Escherichia coli and Salmonella enterica serovar Typhimurium. mBio 12:e00109-21. doi:10.1128/mBio.00109-21 33785633 PMC8092203

[23] Chaban B , Hughes HV , Beeby M . 2015. The flagellum in bacterial pathogens: for motility and a whole lot more. Semin Cell Dev Biol 46:91–103. doi:10.1016/j.semcdb.2015.10.032 26541483

[24] Zhou M , Yang Y , Chen P , Hu H , Hardwidge PR , Zhu G . 2015. More than a locomotive organelle: flagella in Escherichia coli. Appl Microbiol Biotechnol 99:8883–8890. doi:10.1007/s00253-015-6946-x 26346269

[25] Du D , Wang Z , James NR , Voss JE , Klimont E , Ohene-Agyei T , Venter H , Chiu W , Luisi BF . 2014. Structure of the AcrAB-TolC multidrug efflux pump. Nature 509:512–515. doi:10.1038/nature13205 24747401 PMC4361902

[26] Murakami S , Nakashima R , Yamashita E , Matsumoto T , Yamaguchi A . 2006. Crystal structures of a multidrug transporter reveal a functionally rotating mechanism. Nature 443:173–179. doi:10.1038/nature05076 16915237

[27] Murakami S , Nakashima R , Yamashita E , Yamaguchi A . 2002. Crystal structure of bacterial multidrug efflux transporter AcrB. Nature 419:587–593. doi:10.1038/nature01050 12374972

[28] Ma D , Alberti M , Lynch C , Nikaido H , Hearst JE . 1996. The local repressor AcrR plays a modulating role in the regulation of acrAB genes of Escherichia coli by global stress signals. Mol Microbiol 19:101–112. doi:10.1046/j.1365-2958.1996.357881.x 8821940

[29] Kim YJ , Im SY , Lee JO , Kim OB . 2016. Potential swimming motility variation by AcrR in Escherichia coli. J Microbiol Biotechnol 26:1824–1828. doi:10.4014/jmb.1607.07058 27558437

[30] Pérez-Varela M , Corral J , Aranda J , Barbé J . 2019. Roles of efflux pumps from different superfamilies in the surface-associated motility and virulence of Acinetobacter baumannii ATCC 17978. Antimicrob Agents Chemother 63:e02190-18. doi:10.1128/AAC.02190-18 30642939 PMC6395914

[31] Shirshikova TV , Sierra-Bakhshi CG , Kamaletdinova LK , Matrosova LE , Khabipova NN , Evtugyn VG , Khilyas IV , Danilova IV , Mardanova AM , Sharipova MR , Bogomolnaya LM , Gales AC . 2021. The ABC-type efflux pump MacAB is involved in protection of Serratia marcescens against aminoglycoside antibiotics, polymyxins, and oxidative stress. mSphere 6. doi:10.1128/mSphere.00033-21 PMC8546677 33692192

[32] Thota SS , Chubiz LM . 2019. Multidrug resistance regulators MarA, SoxS, Rob, and RamA repress flagellar gene expression and motility in Salmonella enterica serovar Typhimurium. J Bacteriol 201:e00385-19. doi:10.1128/JB.00385-19 31501286 PMC6832076

[33] Li M , Gu R , Su CC , Routh MD , Harris KC , Jewell ES , McDermott G , Yu EW . 2007. Crystal structure of the transcriptional regulator AcrR from Escherichia coli. J Mol Biol 374:591–603. doi:10.1016/j.jmb.2007.09.064 17950313 PMC2254304

[34] Su CC , Rutherford DJ , Yu EW . 2007. Characterization of the multidrug efflux regulator AcrR from Escherichia coli. Biochem Biophys Res Commun 361:85–90. doi:10.1016/j.bbrc.2007.06.175 17644067 PMC2104644

[35] Harmon DE , Ruiz C . 2022. The multidrug efflux regulator AcrR of Escherichia coli responds to exogenous and endogenous ligands to regulate efflux and detoxification. mSphere 7:e0047422. doi:10.1128/msphere.00474-22 36416552 PMC9769551

[36] Lee JO , Cho KS , Kim OB . 2014. Overproduction of AcrR increases organic solvent tolerance mediated by modulation of SoxS regulon in Escherichia coli. Appl Microbiol Biotechnol 98:8763–8773. doi:10.1007/s00253-014-6024-9 25176444

[37] Wang S , Fleming RT , Westbrook EM , Matsumura P , McKay DB . 2006. Structure of the Escherichia coli FlhDC complex, a prokaryotic heteromeric regulator of transcription. J Mol Biol 355:798–808. doi:10.1016/j.jmb.2005.11.020 16337229

[38] Osterman IA , Dikhtyar YY , Bogdanov AA , Dontsova OA , Sergiev PV . 2015. Regulation of flagellar gene expression in bacteria. Biochemistry (Mosc) 80:1447–1456. doi:10.1134/S000629791511005X 26615435

[39] Schaffner SH , Lee AV , Pham MTN , Kassaye BB , Li H , Tallada S , Lis C , Lang M , Liu Y , Ahmed N , Galbraith LG , Moore JP , Bischof KM , Menke CC , Slonczewski JL . 2021. Extreme acid modulates fitness trade-offs of multidrug efflux pumps MdtEF-TolC and AcrAB-TolC in Escherichia coli K-12. Appl Environ Microbiol 87:e0072421. doi:10.1128/AEM.00724-21 34085861 PMC8315180

[40] Gabel CV , Berg HC . 2003. The speed of the flagellar rotary motor of Escherichia coli varies linearly with protonmotive force. Proc Natl Acad Sci U S A 100:8748–8751. doi:10.1073/pnas.1533395100 12857945 PMC166384

[41] Su C-C , Yu EW . 2007. Ligand-transporter interaction in the AcrB multidrug efflux pump determined by fluorescence polarization assay. FEBS Lett 581:4972–4976. doi:10.1016/j.febslet.2007.09.035 17910961 PMC2254335

[42] Duval V , Lister IM . 2013. MarA, SoxS and Rob of Escherichia coli - global regulators of multidrug resistance, virulence and stress response. Int J Biotechnol Wellness Ind 2:101–124. doi:10.6000/1927-3037.2013.02.03.2 24860636 PMC4031692

[43] Alekshun MN , Levy SB . 2007. Molecular mechanisms of antibacterial multidrug resistance. Cell 128:1037–1050. doi:10.1016/j.cell.2007.03.004 17382878

[44] Baba T , Ara T , Hasegawa M , Takai Y , Okumura Y , Baba M , Datsenko KA , Tomita M , Wanner BL , Mori H . 2006. Construction of Escherichia coli K-12 in-frame, single-gene knockout mutants: the Keio collection. Mol Syst Biol 2:0008. doi:10.1038/msb4100050 PMC1681482 16738554

[45] Guzman LM , Belin D , Carson MJ , Beckwith J . 1995. Tight regulation, modulation, and high-level expression by vectors containing the arabinose P_BAD_ promoter. J Bacteriol 177:4121–4130. doi:10.1128/jb.177.14.4121-4130.1995 7608087 PMC177145

[46] Stecher G , Tamura K , Kumar S . 2020. Molecular evolutionary genetics analysis (MEGA) for macOS. Mol Biol Evol 37:1237–1239. doi:10.1093/molbev/msz312 31904846 PMC7086165

[47] Ruiz C , Levy SB . 2010. Many chromosomal genes modulate MarA-mediated multidrug resistance in Escherichia coli. Antimicrob Agents Chemother 54:2125–2134. doi:10.1128/AAC.01420-09 20211899 PMC2863627

[48] Viveiros M , Dupont M , Rodrigues L , Couto I , Davin-Regli A , Martins M , Pagès J-M , Amaral L . 2007. Antibiotic stress, genetic response and altered permeability of E. coli. PLoS One 2:e365. doi:10.1371/journal.pone.0000365 17426813 PMC1838523

